# The Effect of Zinc Sulfate Treatment on Diabetic Cardiomyopathy in an Aged Female Rat Model of Type 2 Diabetes

**DOI:** 10.3390/nu18122005

**Published:** 2026-06-20

**Authors:** Nilufer Akgun-Unal, Omer Unal, Gamze Altun, Elif Gulbahce-Mutlu, Ahmet Akkoca, Mustafa Ayyildiz

**Affiliations:** 1 Department of Biophysics, Faculty of Medicine, Ondokuz Mayis University, 55139 Samsun, Türkiye; 2 Department of Physiology, Faculty of Medicine, Samsun University, 55080 Samsun, Türkiye; omer.unal@samsun.edu.tr; 3 Department of Histology and Embryology, Faculty of Medicine, Ondokuz Mayis University, 55139 Samsun, Türkiye; gamze.altun@omu.edu.tr; 4 Department of Medical Biology, Faculty of Medicine, KTO Karatay University, 42020 Konya, Türkiye; elif.mutlu@karatay.edu.tr; 5 Department of Biophysics, Faculty of Medicine, Necmettin Erbakan University, 42090 Konya, Türkiye; aakkoca@erbakan.edu.tr; 6 Department of Physiology, Faculty of Medicine, Ondokuz Mayis University, 55139 Samsun, Türkiye; mayyildiz@omu.edu.tr

**Keywords:** diabetic cardiomyopathy, aged rats, zinc, isometric contraction, calcium homeostasis, SIRT1, immunohistochemistry

## Abstract

**Background/Objectives:** Diabetic cardiomyopathy (DCM) is largely driven by severe oxidative stress and calcium dyshomeostasis. We examined the targeted antioxidant and therapeutic effects of zinc sulfate (*ZnSO*_4_) on contractile dynamics, oxidative damage, calcium turnover, and apoptosis/fibrosis in aged female rats with type 2 diabetes. **Methods:** Thirty-two aged female Wistar rats were divided into Control, Control + *ZnSO*_4_, Diabetes (DM), and DM + *ZnSO*_4_ groups. DM was induced via high-fat diet and 30 mg/kg streptozotocin. After a 4-week complication period, treatment groups received 10 mg/kg/day *ZnSO*_4_ (i.p.) for 6 weeks. Left ventricular papillary muscle contraction, oxidative/antioxidant markers (MDA/GSH), and gene expressions (SIRT1, GLUT4, SERCA2a, RyR2, Cav1.2, PLN) were evaluated. Myocardial architecture, fibrosis, and apoptosis were analyzed immunohistochemically. In DM rats, contractile force (CF) and velocities (±dF/dtmax) significantly declined. **Results:** Concurrently, SIRT1, GLUT4, SERCA2a, RyR2, Cav1.2, and antioxidant GSH decreased, while oxidative lipid damage (MDA), PLN, Caspase-3 activity, Collagen I, and fibrosis increased (*p* < 0.001). *ZnSO*_4_ treatment in diabetic rats acted as a potent antioxidant modulator; it restored redox balance, activated the SIRT1/GLUT4 pathway, protected calcium-handling proteins from oxidative degradation, and significantly improved contractile dynamics. It also preserved myocardial architecture by reducing apoptosis and fibrosis. In healthy rats, *ZnSO*_4_ caused mild stress and early fibrosis. **Conclusions:** In conclusion, while inducing mild stress in healthy myocardium, zinc supplementation provides robust antioxidant protection in diabetic hearts. It activates SIRT1, suppresses oxidative damage, maintains calcium homeostasis, and restores contractile dynamics, demonstrating strong antioxidant therapeutic potential against DCM.

## 1. Introduction

Diabetes mellitus is a complex, age-related chronic metabolic disorder with a growing global incidence rate [1], and it is associated with a high risk of long-term systemic complications [2]. These chronic vascular complications are generally classified into two main categories: microvascular and macrovascular diseases. Microvascular complications frequently manifest as diabetic nephropathy, neuropathy, and retinopathy. Conversely, macrovascular complications encompass severe systemic conditions such as atherosclerosis, peripheral arterial disease, stroke, and hypertension [3]. While both Type 1 and Type 2 diabetes share these debilitating long-term complications, cardiovascular impairments stand out as the primary cause of morbidity and mortality. Notably, profound structural and functional cardiac abnormalities can develop in diabetic patients even in the complete absence of coronary artery disease, hypertension, or significant valvular disease. This specific and independent manifestation of macrovascular complication is known as diabetic cardiomyopathy, which serves as the primary focus of this study [4]. One of the main causes of illness and death in diabetic patients is diabetic cardiomyopathy, which can occur regardless of factors such as coronary artery disease or high blood pressure [4]. Diabetic cardiomyopathy’s cellular basis involves multifactorial mechanisms including hyperglycemia, oxidative stress, impaired energy metabolism, and calcium homeostasis abnormalities [3,5]. Excessive production of reactive oxygen species (ROS) due to hyperglycemia causes lipid peroxidation and cellular damage in myocardial tissue [2,6]. Increased oxidative stress hampers the proteins essential for coordinating heart muscle contraction and relaxation, including the sarcoplasmic reticulum’s Ca^2+^-pumping enzyme SERCA2a and the ryanodine receptor (RyR2), ultimately predisposing cells to malfunction [7,8].

The aging process increases the risk of developing diabetic cardiomyopathy by causing structural and functional impairments in the cardiovascular system, as reported by Strait and Lakatta in 2012 [9]. Disruption of calcium handling mechanisms in the aging heart at the cellular level is characterized by a decrease in SERCA2a protein expression and activity, which impairs myocardial calcium reuptake and negatively affects diastolic relaxation [9]. Concurrently, age-related increments in mitochondrial dysfunction and ROS production intensify oxidative stress in the heart [10]. Cardiac cellular changes in relation to age, as well as dynamics of contraction and excitation, exhibit striking differences between sexes [11,12]. Contractile function in ventricular myocytes of aged male rats declines with age, whereas these contractile functions are largely preserved in aged female rats, as reported by Howlett (2010) [11]. In aged female rats, L-type calcium current (Cav1.2) decreases with age, but sarcoplasmic reticulum calcium stores are maintained by an increase through a compensatory mechanism, thus preserving cellular contractile function [12]. The aged female rat model has significant potential as a model closely resembling physiological reality for investigating sex-specific cardiac compensatory mechanisms, as demonstrated by this finding [12].

The Sirtuin 1 (SIRT1) protein, a NAD+-dependent deacetylase, is a key molecule that protects the myocardium during aging and diabetes [13]. SIRT1 protects the aging heart from oxidative stress and apoptosis triggered by endoplasmic reticulum stress through regulation of cellular metabolism [14,15]. SIRT1 levels decline with age and diabetes, making the heart susceptible to damage and dysfunction of the glucose transporter GLUT4 implicated in insulin resistance [16,17].

Zinc (Zn), an essential trace element, acts as a cofactor in the activation of antioxidant defense systems and the exertion of insulin-like effects [18,19]. Zinc deficiency is associated with cardiovascular diseases and cellular aging processes, as shown in [20]. The literature suggests that zinc supplementation decreases lipid peroxidation (MDA) and increases antioxidant enzyme capacity (GSH) in diabetic animal models [21]. However, studies comprehensively investigating the mechanistic effects of zinc sulfate (ZnSO_4_) treatment on calcium homeostasis and the anti-aging SIRT1/GLUT4 pathway in aged female diabetic hearts are scarce [22].

This study aims to examine the effects of ZnSO_4_ treatment on isolated papillary muscle isometric contraction dynamics, the gene expression levels of calcium channel proteins (SERCA2a, RyR2, phospholamban, Cav1.2), cellular energy metabolism (SIRT1, GLUT4), and oxidative stress (MDA, GSH) in an aged female rat model of diabetes induced by streptozotocin.

## 2. Materials and Methods

### 2.1. Experimental Animals and Adherence to ARRIVE Guidelines

Approval (Approval No. 2025/46) was obtained from the Ondokuz Mayıs University Local Ethics Committee for Animal Experiments (OMÜ HADYEK) for all the experimental procedures in this study. In vivo studies were conducted in full compliance with the ARRIVE (Animal Research: Reporting of In Vivo Experiments) guidelines. To explicitly address the ARRIVE criteria, a total of thirty-two 18-month-old female Wistar albino rats were used in the experiments. After a 1-week acclimatization period under standard laboratory conditions (12-h light/12-h dark cycle, 21 ± 1 °C, with ad libitum access to water and standard diet), the baseline blood glucose levels and body weights were recorded. The 32 rats were then randomly allocated into four equal groups (*n* = 8 per group) using a computer-generated simple randomization sequence to minimize selection bias. Regarding blinding procedures, while the investigators responsible for daily animal care and interventions were aware of the group allocations due to the distinct physical nature of the high-fat diet and injections, all subsequent functional, biochemical, molecular, and histological outcome assessments were strictly performed by independent investigators who were completely blinded to the experimental groups. Importantly, during the entire 15-week experimental period (1-week acclimatization, 4-week induction, 4-week complication, and 6-week treatment phases), no adverse events requiring euthanasia occurred. Despite the advanced age of the rats (18 months), no treatment-related or age-related mortalities occurred during the study, and all 32 rats (*n* = 8 per group) successfully completed the experimental protocol with a zero attrition rate. Furthermore, the HFD and STZ combination yielded a 100% diabetes induction success rate among the animals assigned to the diabetic groups, and the subsequent complication incubation period resulted in uniform cardiomyopathic features in all untreated diabetic rats.

Furthermore, the entire experimental protocol was designed and executed in strict adherence to the “3Rs” (Replacement, Reduction, and Refinement) principles of animal welfare. Regarding Replacement, the use of a whole-animal in vivo model was deemed strictly necessary, as the complex, systemic, and multi-organ pathophysiology of type 2 diabetic cardiomyopathy cannot be adequately replicated using in vitro or in silico alternatives. In terms of Reduction, the required sample size (*n* = 8 per group) was explicitly determined through an a priori statistical power analysis to ensure the absolute minimum number of animals necessary to achieve adequate statistical power and scientific reproducibility. Importantly, this statistically justified minimum sample size was rigorously reviewed and officially approved by the Local Ethics Committee (Approval No. 2025/46) prior to the commencement of the study, thereby strictly preventing unnecessary animal use. Finally, regarding Refinement, all possible measures were implemented to minimize animal suffering, distress, and lasting harm. These included a 1-week acclimatization period, gentle handling during the STZ and zinc sulfate injections, and the strict use of appropriate anesthesia to prevent any pain during the final sacrifice and tissue harvesting procedures.

Aged female rats (18 months old) were specifically chosen for this experimental model due to their unique, sex-specific cardiac compensatory mechanisms during aging. Previous electrophysiological studies have demonstrated that while aging, male rats exhibit a severe, age-dependent decline in ventricular myocyte contractile function, while aged female rats largely preserve their baseline contractility. This preservation in females is achieved through a compensatory increase in sarcoplasmic reticulum (SR) calcium stores, which counteracts the age-related reduction in L-type calcium currents (Cav1.2). Therefore, aged females represent a highly robust and physiologically relevant model to investigate whether the superimposed metabolic stress of type 2 diabetes overcomes this natural cardioprotective mechanism, and to specifically evaluate the potential of zinc sulfate in modulating these resilient calcium handling pathways [11].

### 2.2. Study Groups and Application Protocols

A total of 32 rats were allocated into four equal groups (*n* = 8 per group). This specific sample size was determined through an a priori statistical power analysis to ensure robust, adequately powered, and conclusive results. The experimental protocol, which involved the establishment of an obesity and type 2 diabetic cardiomyopathy model and a zinc (*ZnSO*_4_) treatment regimen, was detailed separately for the following four groups.

Control Group (CON): The rats in this group, consisting of eight animals, were fed a standard diet throughout the entire experimental period, following a one-week acclimatization phase. These animals were injected intraperitoneally (i.p.) with only the carrier solution, a pH 4.5 citrate buffer, during the diabetes induction period, which is the solvent for streptozotocin (STZ), instead of STZ itself. This group was administered physiological saline (SF) in the same volume via i.p. instead of zinc during the 6-week treatment period that started after the 4-week complication waiting period for the diabetes groups.

Control + Zinc Sulfate Group (CON + ZnSO_4_): This group (*n* = 8) was fed a standard diet concurrently with the control group until the end of the experiment and received citrate buffer via i.p. injection instead of STZ. In contrast to the control group, ZnSO_4_ was administered intraperitoneally at a daily dose of 10 mg/kg for 6 weeks, 7 days a week without interruption, at the end of the 4-week period recognized for the development of complications in diabetic groups to test its therapeutic effects [23].

Diabetes Group (DM): Rats in this group (*n* = 8) were fed a high-fat diet (HFD) for 4 weeks after a 1-week adaptation period to mimic the pathophysiology of type 2 diabetes in humans, establishing an obesity foundation (Table 1) [24]. At the end of the 4-week HFD period, following a 12-h fast, rats were administered a single intraperitoneal (i.p.) dose of 30 mg/kg STZ in freshly prepared citrate buffer [25,26]. Rats with blood glucose levels of 300 mg/dL or higher, as measured from blood drawn 72 h after injection from the tail vein, were classified as diabetic. The diabetic cardiac complications were allowed to set up at a cellular level by maintaining the animals on an HFD for a further 4 weeks [3]. During the transition to the 6-week treatment period, this group received physiological saline (SF) via intraperitoneal injection. Injections of the same volume were made instead of ZnSO_4_.

Diabetes + Zinc Sulfate Group (DM + ZnSO_4_): The eight rats in this group (*n* = 8) were subjected to the same obesity induction protocol—four weeks on a high-fat diet (HFD) (Table 1)—and the same diabetes induction protocol (30 mg/kg intraperitoneal streptozotocin, STZ) as the diabetes (DM) group. After diabetes was confirmed, the rats continued on the HFD for a four-week complication-observation period. At the conclusion of that period, to assess the cardioprotective effect of zinc sulfate (ZnSO_4_), the rats received daily intraperitoneal injections of ZnSO_4_ at 10 mg/kg for six weeks, seven days per week, without interruption [23].

### 2.3. Establishment of the Obesity and Diabetes Model

Unlike Type 1 diabetes models induced by high-dose STZ on a standard diet, human Type 2 diabetes is strongly driven by obesity and lipid dysregulation. Therefore, to accurately mimic the human pathophysiological process of Type 2 diabetic cardiomyopathy (glucolipotoxicity), this study intentionally employed the well-established “gold standard” combination of a high-fat diet (HFD) to induce insulin resistance, followed by a low dose of STZ to induce partial *β*-cell dysfunction [24,27]. After the preparatory phase, the rats assigned to the diabetic groups received a continuous HFD for 4 weeks to establish an obese baseline. At the conclusion of this four-week HFD period, the animals were fasted for 12 h, after which a single intraperitoneal injection of 30 mg/kg STZ, freshly dissolved in citrate buffer (pH 4.5) was administered [25]. Blood glucose was measured from tail-vein samples 72 h after STZ injection, and animals with fasting glucose of 300 mg/dL or higher were included in the study.

### 2.4. Complication Incubation Period and Zinc Administration

To permit the full development of diabetes-related cellular injury and cardiomyopathic complications, rats that were confirmed diabetic after STZ injection were kept in a complication-waiting period of at least 4 weeks [3]. During this period, the animals received only a high-fat diet. After the complications were established, zinc sulfate (ZnSO_4_) was administered intraperitoneally at a dose of 10 mg/kg per day for six consecutive weeks, seven days a week, to the Control + ZnSO_4_ and DM + ZnSO_4_ groups in order to assess its therapeutic effect [21]. Throughout this 6-week treatment period, the DM and DM + ZnSO_4_ groups were continuously maintained on the HFD to sustain the chronic glucolipotoxic stress, whereas the CON and CON + ZnSO_4_ groups were maintained on the standard diet. Zinc sulfate (*ZnSO*_4_) was specifically selected as the zinc donor due to its high aqueous solubility, making it highly optimal and stable for injectable formulations. Although inorganic zinc salts typically exhibit limited oral bioavailability when administered enterally, our utilization of the intraperitoneal (i.p.) administration route completely bypassed the gastrointestinal absorption barrier. This methodological choice ensured rapid and maximal systemic delivery of zinc to the target myocardial tissue. Furthermore, this specific salt and i.p. dosage protocol has been extensively validated in the literature as an effective and safe strategy to induce endogenous cardiac antioxidant mechanisms without provoking heavy metal toxicity [23].

### 2.5. Contraction Records in the Isolated Organ Bath

At the conclusion of the treatment period, the hearts of rats euthanized under anesthesia were promptly excised and placed into a Petri dish containing modified Krebs solution (119 mM NaCl, 4.8 mM KCl, 1.8 mM CaCl_2_, 1.2 mM MgSO_4_, 1.2 mM KH_2_PO_4_, 20 mM NaHCO_3_, 10 mM glucose, pH 7.4). The dish was aerated with 95% O_2_ and 5% CO_2_. Papillary muscles from the left ventricle were positioned in an isolated organ bath; one end was attached to an FT03 force transducer (Grass Instruments, West Warwick, RI, USA) and the other to a micromanipulator. The modified Krebs solution was continuously perfused through the bath at 37 °C [22,28].

Suspended papillary muscles were equilibrated for 30 min under a 2 g preload while receiving 2 ms supramaximal stimuli. Basic contraction parameters were measured using stimuli that did not cause fatigue at a frequency of 0.2 Hz. The stimulation frequency was gradually increased to 1, 2, 3, 4, and 5 Hz, with recordings taken every 5 min to assess the muscles’ frequency-dependent responses [29]. The recordings were digitized using a data acquisition unit (MP36, Biopac Systems Inc., Goleta, CA, USA) running BSL 4.1.1 software. The mass of each muscle preparation was measured in milligrams, and the force of contraction was recorded in grams per second. Contraction force (CF, g), +dF/dtmax (g/s), −dF/dtmax (g/s), and area under the curve (AUC, g·s) were normalized by dividing by the muscle mass in milligrams [30]. A representative schematic diagram detailing the measurement of these evaluated isometric contraction and relaxation parameters from a single muscle twitch is provided in Supplementary Figure S1.

### 2.6. Biochemical and Molecular Analyses in Myocardial Papillary Muscle Tissue

The concentrations of malondialdehyde (MDA) and glutathione (GSH)—an indicator of antioxidant capacity—were measured in papillary muscle tissue samples by ELISA to evaluate myocardial oxidative stress [21]. Furthermore, the mRNA expression levels of SERCA2a, ryanodine receptor (RyR2), L-type calcium channel (Cav1.2), phospholamban, SIRT1, and GLUT4—genes involved in calcium homeostasis and energy metabolism—were quantified using quantitative real-time PCR after total RNA extraction and cDNA synthesis [14,27]. Total RNA was isolated from cardiac tissue using a commercial kit (Bio Basic, Markham, ON, Canada) according to the manufacturer’s protocol. Purity and concentration were assessed spectrophotometrically on a microplate reader (Multiskan SkyHigh Microplate, Thermo Fisher, Waltham, MA, USA), and electrophoresis was used to verify RNA integrity. cDNA was synthesized using a commercial reverse-transcription kit (High Capacity cDNA Reverse Transcription Kit, Catalog No. 4368813, Thermo Fisher, Vilnius, Lithuania) on a ProFlex PCR System (Thermo Fisher, Waltham, MA, USA) according to the manufacturer’s instructions. The primer sequences are listed in Table 2. Thermal cycling was initiated with a pre-incubation step at 95 °C for 10 min to ensure complete template denaturation and polymerase activation, followed by 45 amplification cycles comprising denaturation at 95 °C for 10 s, primer-dependent annealing at 60 °C for 10 s, and extension at 72 °C for 10 s. Fluorescence acquisition was performed in single-acquisition mode at the extension phase of each cycle. To verify the specificity of the PCR amplification and exclude non-specific products or primer-dimer formation, melt curve analysis was subsequently performed using sequential incubation at 95 °C for 10 s and 65 °C for 60 s, followed by a continuous temperature increase to 97 °C with continuous fluorescence monitoring. The protocol was completed with a final cooling step at 40 °C for 30 s. The obtained data were analyzed using the comparative Ct (ΔΔCt) method of relative quantification and prepared for comparison between groups [31].

### 2.7. Histological Analyses

After the experimental procedures were completed and the left ventricular papillary muscles were isolated for the organ bath experiments, the remaining left ventricular mid-myocardial tissues were dissected. These tissues were fixed in a solution containing 2% paraformaldehyde and 2% glutaraldehyde. Following fixation, the tissues were processed in the usual manner and embedded in paraffin using an L-shaped iron to produce paraffin blocks. During embedding, the left ventricular tissues were carefully oriented to obtain both longitudinal and transverse (cross-sectional) planes of the myocardial fibers. Labeling was performed blindly. From the paraffin blocks, sections of 5 µm and 4 µm thickness were cut with a Leica RM2245 microtome (Leica, Nussloch, Germany). For collagen deposition, collagen area fraction (%) was analyzed using ImageJ version 1.53 (National Institutes of Health, Bethesda, MD, USA) with a dotted area scale, after scanning 5 areas from each section at ×40 magnification. The fraction of collagen fibers in the myocardium layer was evaluated [32].

Sections 4 µm thick were used for immunohistochemical analysis. Deparaffinized tissues were treated with 3% hydrogen peroxidase. After washing with phosphate buffer (pH = 7.4), the sections were first treated with citrate buffer (pH = 6.0) in a microwave oven at 850 watts for 3 min, followed by treatment at 170 watts for 17 min, to complete the antigen-retrieval step. Subsequently, sections treated with the protein block were washed again with phosphate buffer (pH = 7.4) and incubated overnight at +4 °C with primary antibodies. Sections were then incubated with a biotin-containing secondary antibody for 10 min, washed with phosphate buffer, and treated with the streptavidin detection system. After the detected reactions were stained with AEC chromogen, reverse staining was performed with Mayer’s hematoxylin. The Mouse and rabbit-specific HRP/AEC Detection IHC kit (AB93705, Abcam, Waltham, MA, USA) was used for immunohistochemical analyses. In the immunohistochemical evaluation, the immunoreactivity of anti-VEGF antibodies (1:200, Sc 7269, Lot B2825, Santa Cruz Biotechnology, Dallas, TX, USA) to assess angiogenesis, anti-TGFβ1 antibodies (1:300, Sc130348, Lot G2125, Santa Cruz Biotechnology) to assess fibrosis, anti-collagen I antibodies (1:300, sc59772, lotC0408, Santa Cruz Biotechnology) to assess cellular stress, and anti-c-Fos antibodies (1:300, sc-52, lot0611, Santa Cruz Biotechnology) and anti-caspase-3 antibodies (1:300, Santa Cruz Biotechnology) to assess apoptosis were semi-quantitatively evaluated in the tissue. Negative controls were performed by omitting the primary antibodies during the incubation step to ensure staining specificity. In this evaluation, 0 indicated no staining; 1 indicated mild staining; 2 indicated moderate staining; and 3 indicated severe staining.

### 2.8. Statistical Analysis

All data obtained from the study are presented as mean ± standard error of the mean (SEM). The suitability of the data for a normal distribution was evaluated using the Shapiro–Wilk normality test. For statistical comparisons among multiple groups, a standard One-Way ANOVA was used for normally distributed parameters, including basal papillary muscle contraction (0.2 Hz), molecular, and biochemical analysis results. Frequency-dependent papillary muscle contraction data and weekly body weight changes were analyzed using a Two-Way repeated measures analysis of variance (Two-way RM ANOVA). The Tukey post hoc test was preferred to identify specific differences between groups following ANOVA. For semi-quantitative histopathological evaluations, which are ordinal data and do not strictly follow a normal distribution, the non-parametric Kruskal–Wallis test was applied, followed by Dunn’s post hoc test. The level of statistical significance was set at *p* < 0.05. All statistical calculations and graph preparation were performed using GraphPad Prism (version 10.0, Boston, MA, USA).

## 3. Results

### 3.1. Body Weight Changes During High-Fat Diet (HFD) Induction

During the initial 4 weeks of the study, the experimental animals’ body weights were recorded weekly to establish a baseline for T2DM, confirm the obesity model, and verify the accuracy of randomization (Figure 1). To ensure the absence of selection bias, data for all four assigned groups were tracked separately from baseline (Initial, W0). No significant difference in body weight was detected between the groups at the outset of the experiment; however, the high-fat diet (HFD)-fed groups (DM and DM + ZnSO_4_) showed a significant increase in body weight over the four-week period compared to the standard diet-fed groups (CON and CON + ZnSO_4_) (*p* < 0.05). This result demonstrates that the HFD protocol effectively induces obesity and metabolic syndrome. Following the confirmation of HFD-induced obesity and the subsequent STZ injection at week 4, routine body weight measurements were concluded to minimize handling-induced stress. During the complication and treatment phases, systemic metabolic progression was instead monitored via periodic fasting blood glucose evaluations (Table 3), aligning with the study’s primary focus on cardiac-specific molecular and functional endpoints. Finally, body weight measurements recorded just prior to sacrifice (Pre-sacrifice) revealed severe weight loss (cachexia) in the untreated DM group, which was significantly lower than the CON group (*p* < 0.05). In contrast, the DM + ZnSO_4_ group exhibited a significant attenuation of this STZ-induced metabolic wasting, with final body weights remaining significantly higher than those of the untreated DM group (*p* < 0.05).

### 3.2. Validation of the Diabetes Model and Glycemic Control

To verify that the high-fat diet (HFD) combined with low-dose streptozotocin (STZ) successfully established a type-2 diabetes model and to assess the systemic impact of zinc therapy, fasting blood glucose was measured at predetermined time points. The readings taken before STZ administration (end of week 4) showed that fasting glucose in all groups remained within the normoglycemic range, and no statistically significant differences were observed between groups (*p* > 0.05).

Measurements taken 72 h after the STZ injection showed a statistically significant rise in blood glucose levels in the rats that received DM induction (DM and DM + ZnSO_4_) compared with the control groups (*p* < 0.001). At this point, animals with blood glucose levels ≥ 300 mg/dL were classified as diabetic, included in the study, and placed in the planned waiting period to allow the development of cardiomyopathic complications. These results confirm that the HFD + STZ protocol resulted in a stable and severe hyperglycemic state.

At the end of the treatment period, the untreated group remained in a severe hyperglycemic state. Conversely, the DM + ZnSO_4_ group, which received ZnSO_4_ supplementation, showed a statistically significant reduction in blood glucose levels compared with the DM group (*p* < 0.05). Zinc supplementation administered to the healthy control group (Control + ZnSO_4_) produced no adverse effect on basal glycemia. Table 3 lists the changes in blood glucose levels observed in each study group over the course of the experiment.

### 3.3. Contraction Parameters

No statistically significant differences were observed in the contraction time (CT) and relaxation time (RT) parameters measured with 0.2 Hz stimuli that do not cause muscle fatigue. In the diabetic (DM) group, the contraction force (CF), maximum relaxation rate (−dF/dt_max_), maximum contraction velocity (+dF/dt_max_), and contraction power (area under the curve, AUC) were all significantly lower. Treatment with ZnSO_4_ mitigated the DM-induced reductions in contraction velocity and power. Additionally, the AUC data showed that zinc treatment preserved the overall work capacity of the papillary muscle. These results demonstrate the therapeutic benefit of Zn in sustaining the functional integrity of papillary muscle in the streptozotocin-induced diabetes model (Table 4).

Figure 2 displays the detailed frequency-dependent muscle contraction parameters. As the stimulation frequency increased from 1 Hz to 5 Hz, the diabetic (DM) group exhibited a progressive and significant decline across all measured parameters. Specifically, the peak Contraction Force (CF) in the DM group was severely depressed, with the most pronounced performance losses observed at the highest frequency tested (5 Hz). Similarly, the maximum rates of contraction (+dF/dt_max_) and relaxation (−dF/dt_max_) were significantly blunted in the diabetic myocardium across all frequencies, indicating severe systolic and diastolic dysfunction under an increased workload. Furthermore, the Area Under the Curve (AUC), representing the total mechanical energy output, was markedly reduced in the DM group. Importantly, zinc supplementation effectively mitigated these functional deficits. All four individual parameters (CF, +dF/dt_max_, −dF/dt_max_, and AUC) were significantly greater in the DM + ZnSO_4_ group compared to the untreated DM group at all corresponding frequencies, particularly at high pacing rates (3, 4, and 5 Hz). These comprehensive findings confirm that ZnSO_4_ preserves the contraction and relaxation dynamics, as well as the total mechanical energy of the cardiac muscle, protecting it against workload-induced failure.

### 3.4. Elisa and qPCR Results

Figure 3 displays the results of the molecular and biochemical analyses of the left ventricular papillary muscle tissue. Across all measured parameters, no statistically significant differences were found between the CON and CON + ZnSO_4_ groups, except for Cav1.2 mRNA expression. MDA concentrations, which indicate lipid peroxidation in cardiac tissue, were significantly higher in the DM group than in the control group (*p* < 0.001). In contrast, the DM + ZnSO_4_ group, which received zinc treatment, exhibited significantly lower MDA levels than the DM group (*p* < 0.001) (Figure 3A). Analysis of GSH levels, a marker of antioxidant capacity in heart tissue, revealed a significant reduction in the DM group compared with the control group (*p* < 0.001). Conversely, GSH concentrations in the DM + ZnSO_4_ group were significantly higher than those in the DM group (*p* < 0.001) (Figure 3B).

Molecular analysis revealed that SIRT1 mRNA expression was markedly decreased in the DM group compared with that in the CON group (*p* < 0.001). In contrast, the DM + ZnSO_4_ group, which received zinc, showed a significant increase in SIRT1 expression compared with the DM group (*p* < 0.001) (Figure 3G). Similarly, GLUT4 mRNA expression was significantly lower in the DM group than in the CON group (*p* < 0.001), and GLUT4 expression was significantly higher in the DM + ZnSO_4_ group than in the DM group (*p* < 0.001) (Figure 3H).

When examining genes involved in calcium homeostasis, SERCA2a mRNA expression was markedly lower in the DM group than in the control group (*p* < 0.001) and significantly higher in the DM + ZnSO_4_ group than in the untreated DM group (*p* < 0.001) (Figure 3C). Cav1.2 mRNA expression was significantly reduced in the CON + ZnSO_4_ group compared with the CON group. Cav1.2 mRNA expression was significantly lower in the DM group than in the CON group (*p* < 0.001), whereas it was significantly higher in the DM + ZnSO_4_ group than in the DM group (*p* < 0.001) (Figure 3F). Conversely, PLN (phospholamban) mRNA expression was significantly elevated in the DM group compared with that in the CON group (*p* < 0.001), and it was significantly reduced in the DM + ZnSO_4_ group compared with that in the DM group (*p* < 0.001) (Figure 3D). Finally, RyR2 mRNA expression was markedly lower in the DM group than in the CON group (*p* < 0.001) and significantly higher in the DM + ZnSO_4_ group than in the DM group (*p* < 0.001) (Figure 3E).

### 3.5. Evaluation of Cardiac Fibrosis

Examination of Masson trichrome-stained sections revealed that in the CON group, the muscle fibers were arranged in parallel, with minimal fibrosis around the vessels supplying the heart. However, in the CON + ZnSO_4_ group, dense collagen deposition and, consequently, dense fibrosis were observed in the myocardial layer. Furthermore, perivascular fibrosis was observed around the coronary vessels in the CON + ZnSO_4_ group. In the DM group, degeneration of muscle nuclei, widespread fibrosis, and inflammation were observed. Additionally, the widespread spacing and gaps between muscle fibers suggest edema. Moreover, widespread perivascular and interstitial fibrosis was observed in some areas of the DM + ZnSO_4_ group. When histomorphometric results for collagen accumulation across groups were evaluated, an increase was observed in the CON + ZnSO_4_ group compared with the DM + ZnSO_4_ and CON groups (*p* = 0.0035 and *p* < 0.0001, respectively). Additionally, an increase was observed in the DM group compared to the CON group (*p* = 0.0039). No differences were observed between the other groups (Figure 4 and Figure 5).

### 3.6. Immunohistochemical Findings

The immunoreactivity of anti-VEGF, anti-TGFβ1, and anti-Collagen I primary antibodies was examined to assess fibrosis and angiogenesis in all groups. Regarding anti-Collagen I expression, a significant increase was observed in the DM group compared to the DM + ZnSO_4_ and CON groups (*p* = 0.0175). There was no difference in anti-Collagen I expression among the other groups (*p* > 0.05). On the other hand, no differences were observed between the groups in terms of anti-VEGF and anti-TGFβ1 primary antibodies (*p* > 0.05) (Figure 6 and Figure 7).

When anti-caspase-3 and anti-c-Fos expression was evaluated in sections from all groups, an increase in anti-caspase-3 activity was observed in the DM group compared to the DM + ZnSO_4_ group (*p* = 0.0051), while an increase was detected in the CON + ZnSO_4_ group compared to both the CON and DM + ZnSO_4_ groups (*p* = 0.0312 and *p* = 0.0017, respectively). No difference was detected between the DM and CON groups (*p* > 0.05). In semi-quantitative evaluations of anti-c-Fos, an increase was found in the CON + ZnSO_4_ group compared to the CON group (*p* = 0.0059) (Figure 6, Figure 7 and Figure 8).

## 4. Discussion

This study examined the functional, molecular, biochemical, and histopathological effects of zinc sulfate (ZnSO_4_) treatment on diabetic cardiomyopathy (DCM) in an aged female rat model of type 2 diabetes induced by a high-fat diet (HFD) and low-dose streptozotocin (STZ). The results show that the severe mechanical dysfunction, disrupted calcium homeostasis, heightened oxidative stress, and cellular apoptosis associated with diabetes in myocardial tissue can be largely reversed by a 6-week ZnSO_4_ supplementation [21,23].

The primary functional marker of diabetic cardiomyopathy is systolic and diastolic dysfunction caused by disturbances in excitation–contraction (EC) coupling [4]. In our isolated organ-bath experiments, the peak contraction force (CF), maximum contraction rate (+dF/dt_max_), and maximum relaxation rate in the papillary muscles of the diabetic (DM) group, as well as the same parameters at the baseline stimulation frequency (0.2 Hz), were significantly lower than those in the control group. A comparable organ-bath study on the diaphragm—a skeletal/respiratory muscle—using an STZ-induced diabetes model showed that diabetes directly impairs muscle mechanics and alters the maximum relaxation rate. MitoTEMPO, a mitochondria-specific antioxidant, restored these deficits to near-control levels [33]. This result confirms the harmful impact of oxidative stress on muscle function. In our own work, the DM + ZnSO_4_ group treated with ZnSO_4_ exhibited a marked improvement in muscle work capacity (AUC) and both contraction and relaxation rates, approaching control values even at higher stimulation frequencies (3–5 Hz).

The pattern of dysfunction and recovery observed in the mechanical recordings matches the molecular expression data for calcium-handling proteins. Normal systolic contraction depends on L-type calcium channels (Cav1.2), which allow calcium to enter the cell, and ryanodine receptors (RyR2), which release calcium from the SR [34]. Our molecular analysis showed that both Cav1.2 and RyR2 were markedly down-regulated in the DM group. This reduction explains the low calcium-release rate (+dF/dt_max_) observed in the papillary muscle. Conversely, the expression of SERCA2a, which pumps calcium back into the SR during diastole, is decreased, and the levels of its inhibitor phospholamban (PLN) are higher in the diabetic heart. This combination blocks calcium reuptake and accounts for the slowed relaxation rate (−dF/dt_max_) [7,35]. Zinc treatment (DM + ZnSO_4_) restored the calcium cycle by lowering PLN expression and normalizing SERCA2a, RyR2, and Cav1.2 levels demonstrated that free cytosolic zinc can directly alter the open probability of cardiac RyR2 channels. Thus, zinc exerts its beneficial effects on calcium proteins by re-establishing redox balance and directly modulating channels such as RyR2 [36,37].

Severe cellular oxidative stress caused by glucolipotoxicity is the primary pathological factor driving the observed molecular disruption in the diabetic heart. During the progression of diabetic cardiomyopathy, chronic hyperglycemia and glucolipotoxicity lead to excessive reactive oxygen species (ROS) production, predominantly originating from mitochondrial respiratory chain dysfunction [10]. When this sustained ROS generation overwhelms endogenous antioxidant defenses, it induces severe lipid peroxidation, evidenced by elevated MDA levels, and causes direct structural damage to cellular membranes [21]. Furthermore, chronic oxidative stress severely impairs excitation–contraction coupling by directly oxidizing critical calcium-handling proteins. Specifically, ROS-mediated damage reduces SERCA2a activity and promotes ryanodine receptor (RyR2) calcium leakage, which disrupts intracellular calcium compartmentalization and contributes directly to contractile dysfunction [34,36]. Beyond functional impairment, elevated ROS levels alter mitochondrial membrane permeability and trigger pro-apoptotic signaling cascades. This is characterized by the upregulation of the pro-apoptotic protein Bax and Caspase-3 activation, alongside the suppression of the anti-apoptotic protein Bcl-2 [21]. Ultimately, this progressive oxidative environment stimulates profibrotic pathways, accelerating collagen deposition and interstitial fibrosis, which collectively drive adverse myocardial remodeling in the diabetic heart [3]. Our results show that MDA levels—a marker of lipid peroxidation—have increased sharply in the DM group, whereas GSH reserves, which indicate endogenous antioxidant capacity, have been depleted. Zinc supplementation reestablished redox equilibrium in the diabetic myocardium, markedly reducing MDA levels and elevating GSH levels [21]. The Sirtuin 1 (SIRT1) pathway plays a key role in antioxidant defense and metabolic regulation. Zinc treatment significantly increases SIRT1 and GLUT4—the insulin-dependent glucose transporter—whose expression is suppressed in the diabetic group. These findings suggest that zinc functions both as an antioxidant and a metabolic regulator, restoring cellular energy metabolism and insulin sensitivity through the activation of the SIRT1 pathway [14,38]. Furthermore, the systemic metabolic outcomes presented in Table 3 reveal that *ZnSO*_4_ supplementation significantly reduced fasting blood glucose levels in the diabetic group (DM + ZnSO_4_). This hypoglycemic effect can be primarily attributed to the insulin-mimetic properties of zinc and its critical role in modulating cellular energy metabolism [19]. In type 2 diabetes, peripheral insulin resistance severely impairs glucose uptake. As demonstrated by our molecular results, zinc treatment significantly upregulated the expression of SIRT1 and the insulin-dependent glucose transporter GLUT4, which were initially suppressed by glucolipotoxicity. The zinc-mediated activation of the SIRT1 axis enhances insulin sensitivity and facilitates the translocation of GLUT4 to the cell membrane, thereby promoting intracellular glucose uptake [15]. Consequently, by breaking systemic insulin resistance and restoring cellular glucose utilization, zinc supplementation effectively clears excess glucose from the circulation, explaining the significant glycemic improvement observed in our diabetic model [21].

Our study’s histopathological, molecular, and functional data clearly show that zinc has a dual, cell-state- and dose-dependent effect on myocardial tissue. In the healthy control group receiving exogenous zinc (CON + ZnSO_4_), we found a marked reduction in Cav1.2 mRNA, an increase in c-Fos immunoreactivity—an early marker of cellular stress—and mild interstitial fibrosis. The observed decrease in contractile force was primarily driven by cellular stress and Cav1.2 suppression. When added to healthy cardiomyocytes that are not zinc-deficient, excess zinc competes with calcium, directly inhibiting calcium entry through L-type calcium channels and reducing systolic contractility [36,39]. High intracellular zinc levels provoke cytotoxic stress by inducing mitochondrial dysfunction and ATP depletion [39]. Akgun-Unal et al. (2025) showed that high-dose zinc disrupts calcium homeostasis in healthy papillary muscles, thereby lowering contractile force [22]. Thus, zinc supplementation—already within physiological limits in healthy tissue—weakens contraction by blocking Cav1.2-mediated calcium influx and triggers cellular stress (elevated c-Fos), leading to early interstitial fibrosis in the myocardium. However, the findings differ markedly in the DM group, which shows severe oxidative stress and zinc deficiency. Histomorphometric analyses of collagen area fraction (%) and immunohistochemical evaluations revealed distinct, state-dependent effects of zinc on myocardial remodeling. In the healthy aged myocardium (CON + ZnSO_4_ group), supranutritional zinc administration induced early extracellular matrix accumulation and cellular stress, evidenced by a significantly increased general collagen area fraction and elevated c-Fos expression (*p* = 0.0059). This suggests that excess zinc in a non-diabetic heart may trigger mild cytotoxic stress and compensatory collagen synthesis via c-Fos, completely independent of the TGF-β1 pathway (which remained statistically unchanged across all groups, *p* > 0.05). Conversely, in the diabetic myocardium (DM group), we observed a significant increase in specific pathological fibrosis, marked by elevated Collagen I expression, driven by severe glucolipotoxicity. Although the zinc-mediated reduction in overall interstitial collagen area fraction (Masson’s trichrome) in the DM + ZnSO_4_ group did not reach statistical significance, zinc treatment significantly suppressed the pathological Collagen I accumulation (*p* = 0.0175) and Caspase-3-mediated apoptosis (*p* = 0.0051). In other words, in the zinc-deficient diabetic heart, zinc supplementation predominantly acts to halt apoptotic cell loss and prevent specific pathological fibrotic remodeling (Collagen I), bypassing the classical TGF-β1 pathway. A distinct apoptotic process occurred in the diabetic myocardium due to increased glucolipotoxicity, and Caspase-3 activation rose. At the same time, although anti-collagen I antibody levels—a fibrosis marker—increased in the DM group, TGF-β1 expression did not change significantly [27,40]. When these results are considered together with the apoptosis data, they strongly indicate that the lost cells are replaced by collagen fibers (replacement fibrosis) after cardiomyocyte death in the diabetic group. Zinc therapy in diabetic tissue (DM + ZnSO_4_) is highly protective against DM.

The treatment lowered apoptotic Caspase-3 activation in the diabetic heart, reduced perivascular/interstitial degeneration, and maintained myocardial architecture [23]. Moreover, the zinc-mediated restoration of cellular energy metabolism via the GLUT4/SIRT1 pathway directly supports this structural preservation. In conclusion, while zinc can cause mild cytotoxic stress by competing with calcium channels in a healthy heart, in a diabetic heart (DM + ZnSO_4_) that has suffered severe oxidative damage, it stops apoptotic cell loss and preserves tissue structure by correcting zinc deficiency.

## 5. Conclusions

Overall, ZnSO_4_ treatment in an aged female rat model of type 2 diabetes stopped lipid peroxidation (MDA) by upregulating SIRT1 and supporting GSH. This reestablishment of redox balance and the zinc-mediated modulation of ion channels maintained the activity of calcium-homeostasis proteins (SERCA2a, RyR2, Cav1.2, and PLN), reduced cellular apoptosis (Caspase-3), and ultimately showed strong therapeutic promise against DKM by restoring the contraction–relaxation dynamics of diabetic myocardial papillary muscle. The limited comparison with similar experiments in this discussion is due to a scarcity of comparable studies in the literature. The majority of preclinical investigations on diabetic cardiomyopathy and zinc supplementation rely on young, male, and Type 1 diabetic animal models. In contrast, the present study specifically utilizes an aged, female, and high-fat-diet-induced Type 2 diabetes model. Because age and sex significantly alter myocardial calcium handling, excitation–contraction coupling, and physiological responses [11,12], direct comparisons with the existing literature are restricted. Furthermore, to our knowledge, the evaluation of isometric contraction dynamics in isolated papillary muscles within this specific demographic is largely unprecedented. The lack of prior publications combining this specific experimental model with direct functional contraction parameters underscores the scientific value of this study, as it provides data for a clinically relevant but underrepresented demographic in cardiovascular research.

## Figures and Tables

**Figure 1:**
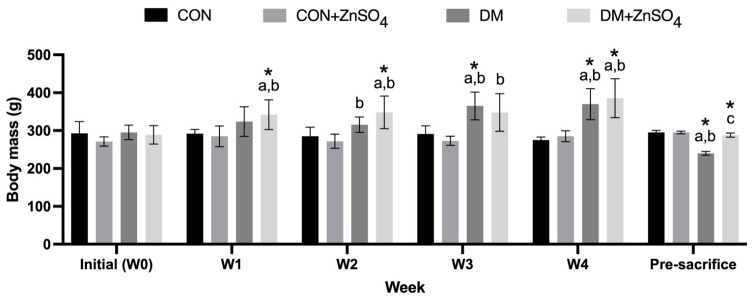
Changes in body weight of the experimental groups. Body weight changes were monitored weekly during the initial 4-week high-fat diet (HFD) induction period (Initial to W4) and immediately prior to sacrifice (Pre-sacrifice). The suitability of the data for a normal distribution was analyzed using the Shapiro–Wilk normality test. Body weight changes were evaluated using a two-way repeated measures analysis of variance (Two-way RM ANOVA) to determine differences over time (repeated measurements) and between groups. The Tukey test was applied as a post hoc test for pairwise comparisons within and between groups. Letters (a, b, c) indicate statistical significance between groups at the respective time point (^a^ vs. CON, ^b^ vs. CON + ZnSO_4_, and ^c^ vs. DM; *p* < 0.05). The (*) symbol indicates a statistically significant difference compared to the previous measurement of the respective group (*p* < 0.05). Data are presented as mean ± SEM (*n* = 8).

**Figure 2:**
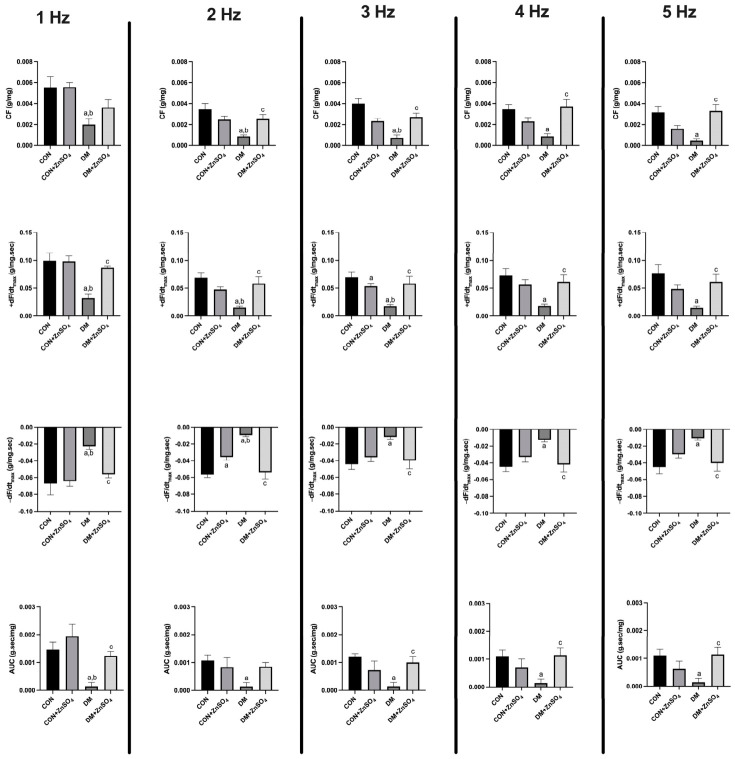
Intergroup comparison of parameters obtained from the frequency protocol of isometric contraction recordings of rat myocardial papillary muscles. *p*-values indicate the results of a one-way ANOVA (repeated measures), while letters on the columns indicate statistical significance according to the Tukey post hoc test. ^a^ vs. CON, ^b^ vs. CON + ZnSO_4_, ^c^ vs. DM indicate statistical significance at the *p* < 0.05 level. Data are presented as mean ± SEM (*n* = 8).

**Figure 3:**
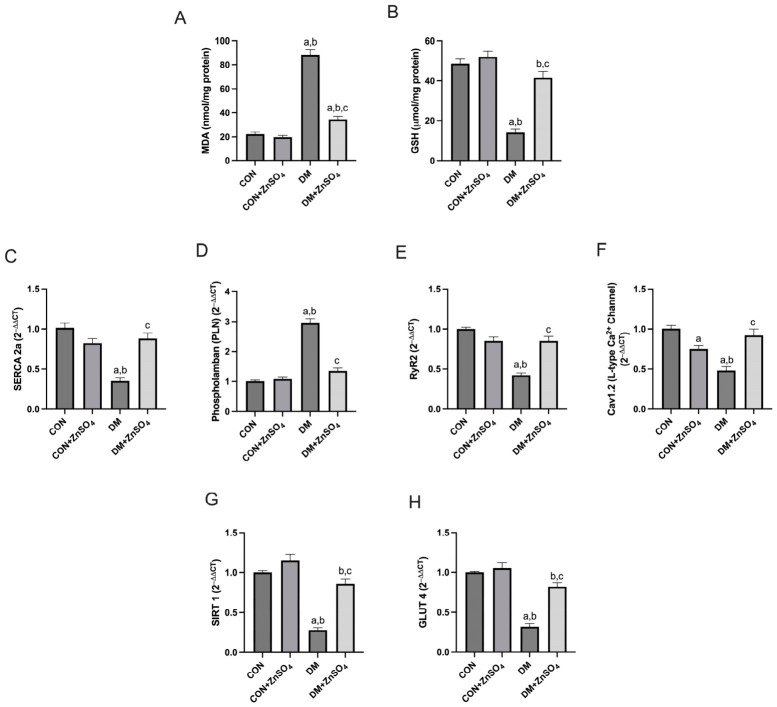
Expression levels of genes associated with oxidative stress markers, calcium homeostasis, and energy metabolism in rat left ventricular papillary muscle tissue. (**A**) MDA, (**B**) GSH, (**C**) SERCA2a, (**D**) Phospholamban (PLN), (**E**) RyR2, (**F**) Cav1.2, (**G**) SIRT1, and (**H**) GLUT4 mRNA expression levels. The obtained molecular data were normalized against the GAPDH reference gene. *p*-values indicate the results of a one-way ANOVA, while the letters on the columns indicate statistical significance based on the Tukey post hoc test. ^a^ vs. CON, ^b^ vs. CON + ZnSO_4_, ^c^ vs. DM indicate statistical significance at the *p* < 0.05 level. Data are presented as mean ± SEM (*n* = 8).

**Figure 4:**
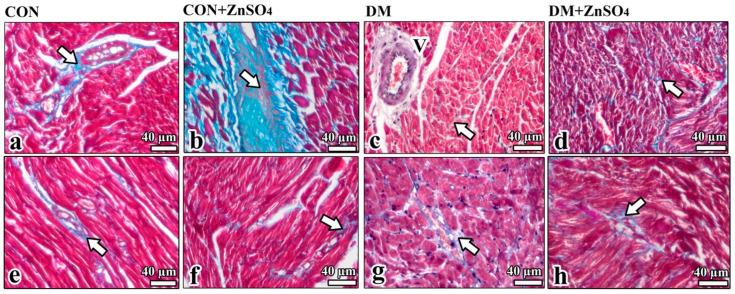
(**a**–**h**) The histomorphology of myocardial muscle from all groups is shown in sections stained with Masson’s trichrome. (**a**,**e**) In the CON group, the myocardial fibers are arranged in parallel. Significant inflammation and fibrosis are absent, and the vessels and heart wall appear normal. (**b**,**f**) In the CON + ZnSO_4_ group, dense collagen is thought to be present. (**c**,**g**) In the images of the DM group, a fibrotic area in the interstitial space is noteworthy. The separation between myocardial fibers is also noticeable. Furthermore, the cellular density observed around the vessels indicates severe inflammation, and distinct vacuolar degeneration is clearly visible in the tissue. (**d**,**h**) In the DM + ZnSO_4_ group, the muscle fiber organization in the myocardial layer is generally preserved, but disruptions in organization persist in some areas. The arrows in all subfigures indicate areas of collagen deposition. Scale bar: 40 µm; V: vessel.

**Figure 5:**
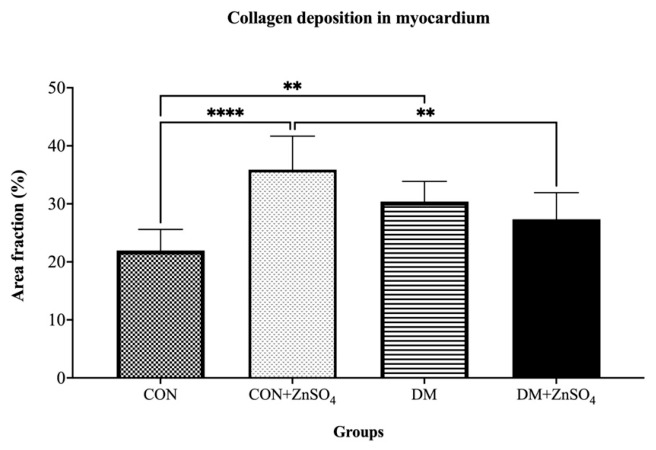
Graph showing the area fraction (%) of collagen deposition in the myocardium for all groups. All data are expressed as mean ± SD. ** *p* < 0.05; **** *p* < 0.0001.

**Figure 6:**
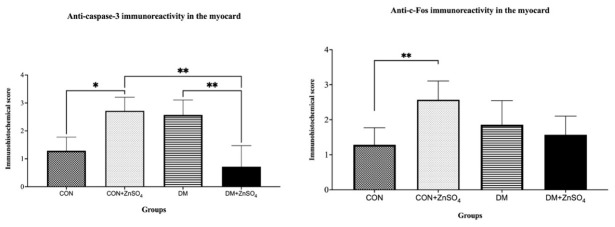
Immunohistochemical scoring graphs of anti-caspase-3 and anti-c-Fos antibodies for all groups. All data are expressed as mean ± SD.* *p* < 0.05; ** *p* < 0.01.

**Figure 7:**
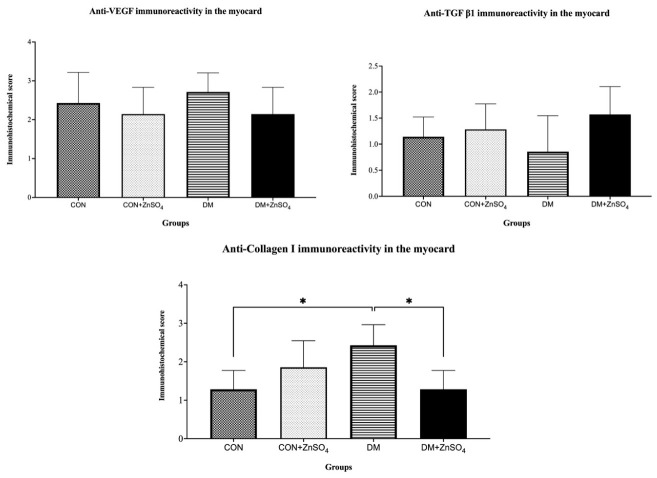
Immunohistochemical scoring graphs of anti-VEGF, anti-TGF-β1, and anti-Collagen I antibodies for all groups. All data are expressed as mean ± SD.* *p* < 0.05.

**Figure 8:**
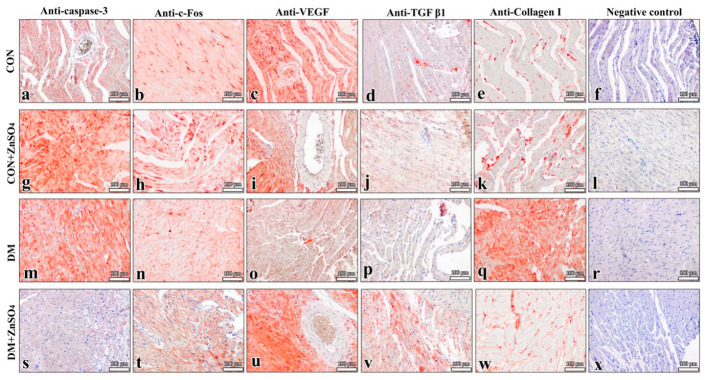
(**a**–**x**) Light microscopic images of anti-caspase-3, anti-c-Fos, anti-VEGF, anti-TGFβ1, and anti-Collagen I immunoreactivity for all groups. Positive reactions appear brown or brick red. (**f**,**l**,**r**,**x**) Negative controls are observed for all stainings, where primary antibodies were omitted. Mayer’s hematoxylin was used for counterstaining. Myocardial staining was examined in all groups. (**a**,**g**,**m**,**s**) Anti-caspase-3 immunoreactivity showed high staining intensity in both the DM and CON + ZnSO_4_ groups, but was significantly suppressed in the DM + ZnSO_4_ group. (**b**,**h**,**n**,**t**) In the groups evaluated for anti-c-Fos immunoreactivity, the most dominant staining was observed in the CON + ZnSO_4_ group. (**c**,**i**,**o**,**u**) Anti-VEGF expression was predominantly stained in all groups. (**d**,**j**,**p**,**v**) Anti-TGF-β1 immunoreactivity showed very low staining intensity across all groups. (**e**,**k**,**q**,**w**) Anti-Collagen I immunoreactivity was significantly increased in the DM group and effectively suppressed by zinc treatment in the DM + ZnSO_4_ group. Scale bar: 100 µm.

**Table 1:** High-fat diet with 60% of energy from fat.

Nutritional Components	g (%)	kcal
Protein	24	18
Carbohydrates	30	22
Fat	35	60
Feed Content	g (grams)	kcal/kg
Casein, 90 Mesh	182.72	730.88
L-Cystine DL-methionine	3	12
Corn Starch	61.08	244.32
Maltodextrin	100	400
Sucrose	50	200
Cellulose, BW200	50	0
Corn oil	25	225
Vegetable oil	245	2205
Mineral Mix S10026	10	0
Dicalcium Phosphate	13	0
Calcium Carbonate	5.5	0
Potassium Citrate, 1 H_2_O	16.5	0
Vitamin Mix V10001	10	40
Choline chloride	2	0
FD&C Dye	0.05	0
Total	773.85	4057.2

**Table 2:** Primers used for qPCR.

Gene	Forward Primer Sequence (5′→3′)	Reverse Primer Sequence (5′→3′)
SERCA2a	CAGAGAGACGCCTGCTTAAAT	GTTCACACCATCACCAGTCATA
Phospholamban (PLN)	GCTCCCAGACTTCACACAACT	GTCTCCTTTTAGGAGGCCTTGG
RyR2	GTCTGGGTGGGCTGGATTAC	CTGCGTTTGATGCTCTCGTG
Cav1.2	CAATCACCGAGGTACACCCAG	GATGCGGGAGTTCTCCTCTG
SIRT1	CATAGGTTAGGTGGCGAGTATG	GTTGGTGGCAACTCTGATAAATG
GLUT4	GCCGGGACACTATACCCTATTC	GGGGGTTCCCCATCTTCAGAG
GAPDH	AACTCCCTCAAGATTGTCAGCAA	GGCATGGACTGTGGTCATGA

**Table 3:** Blood Glucose Levels of the Experimental Groups During the Study Period (mg/dL).

Experimental Groups	Basal (Pre-STZ/Week 4)	Diabetes Induction (72 h Post-STZ)	End of Experiment (Post-Treatment)
CON	98.4 ± 4.2	102.1 ± 5.6	105.8 ± 4.8
CON + ZnSO_4_	96.2 ± 3.8	95.8 ± 4.1	94.5 ± 5.2
DM	104.5 ± 5.1	471.37 ± 23.4 ^a,b^	412.3 ± 22.5 ^a,b^
DM + ZnSO_4_	101.8 ± 4.6	584.5 ± 8.9 ^a,b,c^	215.4 ± 14.8 ^a,b,c^
*p*	0.5730	<0.0001	<0.0001

^a^ vs. CON, ^b^ vs. CON + ZnSO_4_, ^c^ vs. DM indicate statistical significance at the *p* < 0.05 level. Data are presented as mean ± SEM (*n* = 8).

**Table 4:** Basic (0.2 Hz) contraction parameters.

	CON (*n* = 8)	CON + ZnSO_4_ (*n* = 8)	DM (*n* = 8)	DM + ZnSO_4_ (*n* = 8)	*p*
Contraction Time (s)	0.1190 ± 0.0044	0.0946 ± 0.0093	0.1120 ± 0.0037	0.0964 ± 0.0113	0.1026
Relaxation Time (s)	0.1744 ± 0.0066	0.1723 ± 0.0110	0.1843 ± 0.0089	0.1581 ± 0.0173	0.4765
Contraction Force (g/mg)	0.0061 ± 0.0009	0.0066 ± 0.0004	0.0024 ± 0.0005 ^a,b^	0.0032 ± 0.0007 ^a,b^	0.0002
+dF/dt_max_ (g/s·mg)	0.0990 ± 0.0143	0.1126 ± 0.0072	0.0304 ± 0.0033 ^a,b^	0.0841 ± 0.0047 ^c^	<0.0001
−dF/dt_max_ (g/s·mg)	−0.0750 ± 0.0112	−0.0719 ± 0.0035	−0.0246 ± 0.0034 ^a,b^	−0.0475 ± 0.0094	0.0003
Area Under the Curve (g.s/mg)	0.0013 ± 0.0002	0.0020 ± 0.0004	0.0001 ± 0.0001 ^a,b^	0.0013 ± 0.0002 ^c^	0.0006

*p*-values indicate the results of the one-way ANOVA, while the letters on the columns indicate statistical significance based on the Tukey post hoc test. ^a^ vs. CON, ^b^ vs. CON + ZnSO_4_, ^c^ vs. DM indicate statistical significance at the *p* < 0.05 level. The ‘*p*’ column represents the overall One-Way ANOVA *p*-value. Data are presented as mean ± SEM (*n* = 8).

## Data Availability

The data presented in this study are available on request from the corresponding author. The data are not publicly available due to institutional data-management restrictions and ongoing related research.

## Supplementary Materials

The following supporting information can be downloaded at https://www.mdpi.com/article/10.3390/nu18122005/s1, Figure S1: Representative schematic diagram of an isolated papillary muscle isometric contraction (twitch) curve and evaluated mechanical parameters. A schematic illustration detailing the measurement of isometric contraction and relaxation parameters obtained from the isolated left ventricular papillary muscles. The curve represents a single muscle twitch elicited by electrical field stimulation in the isolated organ bath system. CF: Contraction force (peak tension); +dF/dtmax: Maximum rate of contraction (steepest upward slope of the curve); -dF/dtmax: Maximum rate of relaxation (steepest downward slope of the curve); AUC: Area under the curve (total mechanical work/power); CT: Contraction time (time from the onset of twitch to peak tension); RT: Relaxation time (time from peak tension to baseline). To prevent methodological misconceptions and to ensure a fair scientific comparison among experimental groups, raw absolute force values (g) were not used. Instead, all evaluated force-dependent parameters (CT, RT, CF, +dF/dt_max_, −dF/dt_max_, and AUC) were systematically normalized to the wet weight of the respective isolated muscle strip (mg), thereby eliminating the inevitable variations in dissected tissue mass.
