# Supplementary material for: The Effect of Zinc Sulfate Treatment on Diabetic Cardiomyopathy in an Aged Female Rat Model of Type 2 Diabetes
Source: Nutrients. 2026 Jun 20;18(12):2005. doi: 10.3390/nu18122005 (PMC13305182; doi:10.3390/nu18122005)
Supplement: Supplementary file 1 [file nutrients-18-02005-s001.zip › nutrients-4373531-supplementary.pdf]

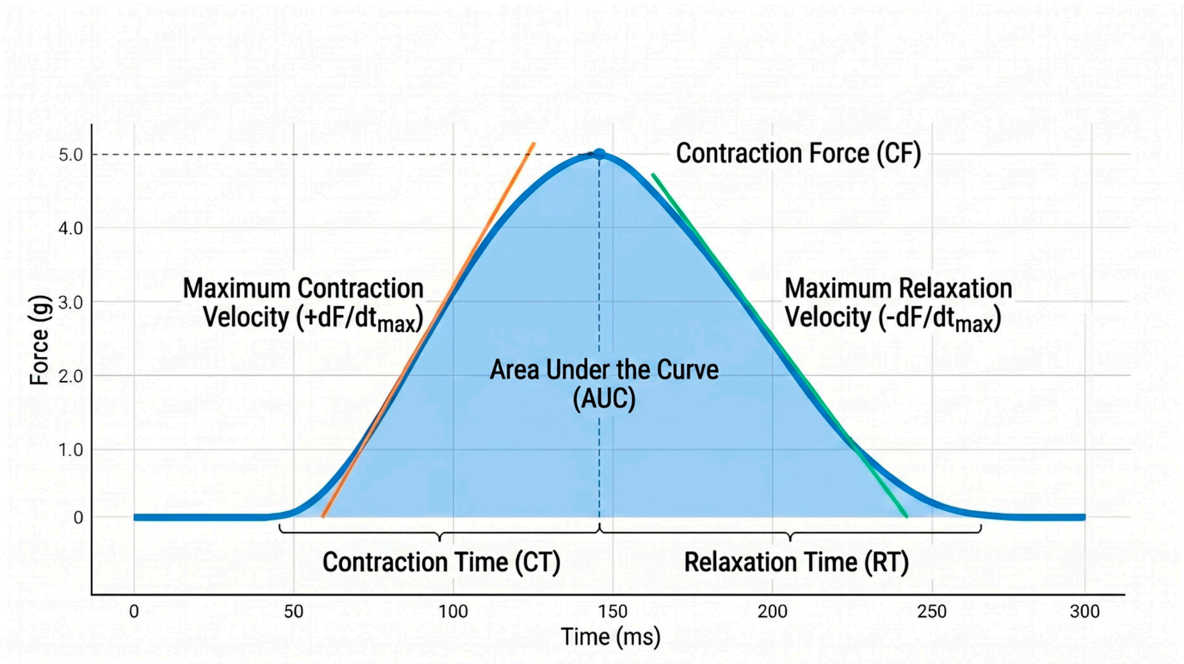

Supplementary Figure S1. Representative schematic diagram of an isolated papillary muscle isometric contraction (twitch) curve and evaluated mechanical parameters. A schematic illustration detailing the measurement of isometric contraction and relaxation parameters obtained from the isolated left ventricular papillary muscles. The curve represents a single muscle twitch elicited by electrical field stimulation in the isolated organ bath system. **CF:** Contraction force (peak tension); **+dF/dtmax:** Maximum rate of contraction (steepest upward slope of the curve); **-dF/dtmax:** Maximum rate of relaxation (steepest downward slope of the curve); **AUC:** Area under the curve (total mechanical work/power); **CT:** Contraction time (time from the onset of twitch to peak tension); **RT:** Relaxation time (time from peak tension to baseline). To prevent methodological misconceptions and to ensure a fair scientific comparison among experimental groups, raw absolute force values (g) were not used. Instead, all evaluated force-dependent parameters (CT, RT, CF, $+dF/dt_{max}$, $-dF/dt_{max}$, and AUC) were systematically normalized to the wet weight of the respective isolated muscle strip (mg), thereby eliminating the inevitable variations in dissected tissue mass.
